# Electro-optical measurement of intense electric field on a high energy pulsed power accelerator

**DOI:** 10.1038/s41598-021-89851-8

**Published:** 2021-05-21

**Authors:** Israel Owens, Chris Grabowski, Andrew Biller, Ben Ulmen, Nathan Joseph, Ben Hughes, Sean Coffey, Debra Kirschner, Ken Struve

**Affiliations:** Sandia National Laboratories, Albuquerque, 87123 USA

**Keywords:** Energy science and technology, Optics and photonics, Physics

## Abstract

We describe a direct electro-optical approach to measuring a strong 118 MV/m narrow pulse width (~ 33 ns) electric field in the magnetically insulated transmission line (MITL) of a pulsed power accelerator. To date, this is the highest direct external electric field measured electro-optically in a pulsed power accelerator, and it is between two to three orders of magnitude higher than values reported in comparable high energy scientific experiments. The MITL electric field is one of the most important operating parameters in an accelerator and is critical to understanding the properties of the radiation output. However, accurately measuring these high fields using conventional pulsed power diagnostics is difficult due to the strength of interfering particles and fields. Our approach uses a free-space laser beam with a dielectric crystal sensor that is highly immune to electromagnetic interference and does not require an external calibration. Here we focus on device theory, operating parameters, laboratory and pulsed power accelerator experiments as well as challenges that were overcome in the measurement environment.

## Introduction

Electro-optical based sensors are ideal for noninvasive measurement of very high electric field strength because they are immune to many issues associated with conventional diagnostics. Sensors such as D-dot probes^1^ measure derivative responses requiring integration or other non-linear post-processing of the sensor signal to obtain a measurement. In contrast, with an electro-optical sensor (EOS) there is a direct linear relationship between the optical signal from the sensor and the electric field to be measured, and the EOS response does not require external calibration or mathematical integration of the signal. An EOS typically utilizes dielectric materials to transmit, sense and receive optical signals to and from a remote data acquisition system. The galvanic isolation inherent with dielectric materials is important as metallic based sensing elements such as the conventional D-dot probes are more prone to noise, improper electrical impedance matching configurations and signal reflections.

To date, the vast amount of literature on electro-optical devices has primarily focused on electro-optical crystal materials and applied voltage levels required for light switching applications^2^, and not measurement of the driving electric field. When the driving field is discussed, existing papers are mostly limited to low DC electric field measurement or low to moderate repetition rate measurement of AC electric fields^3,4^. Fiber optical cables can be used as electric field sensors^5^ but fail at high field strength environments where particle interaction with the fiber material can attenuate and dilute the desired signal through radiation darkening and fluorescence. There is a paucity of papers on high field narrow pulse width electric field measurements using bulk EOS crystals^6,7^. Several experiments have been performed where bulk EOS crystals have been used for electron beam position monitoring in accelerators, but not explicit measurement of electric field components^8–11^. In Consoli^12^ and Robinson et al.^13^, the authors describe electro-optical measurements of electromagnetic pulses generated by laser-plasma interaction in the nanosecond regime where they measured a maximum external electric field^12^ of 261 kV/m. A comprehensive review of various approaches to measure electric fields is given in Peng et al.^14^.

In this paper, we report on an experiment to electro-optically measure the narrow-pulse 118 MV/m external electric field inside the MITL of the High Energy Radiation Megavolt Electron Source III (HERMES III or HIII) pulsed power accelerator^15^ at Sandia National Laboratories. This is one of the highest external electric fields measured electro-optically in a pulsed power accelerator and between two to three orders of magnitude higher than the external field reported in the aforementioned study of laser-plasma interactions^12^. Theory, numerical calculations, a laboratory benchtop experiment with a lithium niobate crystal EOS and conventional diagnostic monitors are highlighted to describe and compare to the HERMES III accelerator experiment data and results. Environment challenges associated with placing a bulk lithium niobate crystal in a high field and particle vacuum environment in the MITL of HERMES III are also treated.

## Methods

### EOS device theory and physical model

In order to determine the strength of the electric field in the HERMES III MITL using an electro-optical approach, we describe the physical process using the Pockel’s effect. By the Pockel’s effect^2^, the polarization of a light beam propagating through an electro-optical crystal will change linearly in proportion to the applied electric field. By placing a pair of crossed linear polarizers on both sides of the electro-optical crystal to equally excite vertical and horizontal optical modes in the crystal relative to the optical axis, the polarization rotation can be transformed into a modulation of optical field intensity incident on a photodetector. A quarter waveplate optical component is added between the linear polarizers to offset the natural birefringence of the electro-optical crystal that skew the signal reference established by the cross polarization. With a voltage V_a_ applied across an approximate flat planar region separated by a distance s and neglecting any edge effects or surface curvature, the rotation in polarization angle Δθ_p_ can be written as^3^:

1 $${\Delta \theta }_{p}=\frac{\pi L{n}_{o}^{3}{r}_{22}{V}_{a}}{\lambda {E}_{3}s}$$

If we consider the benchtop laboratory and the HERMES III experiment parameters where L = 10 mm is the length and d = 6 mm is the thickness of the electro-optical crystal, n_o_ = 2.32 is the ordinary index of refraction, r_22_ = 6.8 pm/V is the electro-optical coefficient in lithium niobate^16^, λ = 532 nm is the laser wavelength, E_3_ is an electric field scaling term^13,17^ that depends on the bulk dielectric constant (ε = 85) and aspect ratio (L/d) of the crystal, s_lab_ = 0.665 cm and s_HIII_ = 14 cm are the distances between the conducting surfaces in the benchtop laboratory and in the HERMES III experiment, respectively and V_lab_ = 4.2 kV and V_HIII_ = 16.25 MV are the applied voltage biases for the laboratory and HERMES III experiments, respectively, then the expected results for the polarization shifts in the laboratory and HERMES III experiments are Δθ_lab_ = 19.5^◦^ and Δθ_HIII_ = 179.0^◦^ upon converting radians to degrees.

The transmission η_c_ of the optical light field through the crossed polarizers and crystal based on the shift in polarization angle Δθ_p_ can be written as:

2 $${\eta }_{c}={\mathrm{sin}}^{2}[{\Delta \theta }_{p}+{ \Delta \theta }_{QWP}]$$

where Δθ_QWP_ is a quarter wave plate polarization angle adjustment to offset the natural crystal birefringence. Using the relevant values for the shift in polarization we find the transmission of the optical light field η_lab_ = 2.9% and η_HIII_ = 99.0%. A complete model expression^7^ that relates the applied conducting surface voltage to the expected output voltage of a silicon photodetector can be written as:

3 $${V}_{m}={P}_{i}{\eta }_{c}{{\eta }_{o}{\eta }_{e}D}_{r}{D}_{i}{D}_{g}$$

where P_i_ = 125 mW is the laser power, η_o_ = 10.20 dB and 12.50 dB are the optical attenuation values in the benchtop laboratory and HERMES III experiment, respectively, η_e_ = 0.2 dB is electrical attenuation in the coaxial cable connectors, D_r_ = 0.195 A/W is the silicon photodetector responsivity at 532 nm, D_i_ = 50 Ω is the detector impedance and D_g_ = 15 is the gain factor of the detector’s internal low noise amplifier. The optical attenuation is primarily from losses incurred through fiber optical cables and associated optical components. For the electrical signal attenuation, the loss value was obtained by directly connecting the silicon photodetector to the oscilloscope.

From the calculation, the expected modulation peak voltage amplitude is 46.21 mV for the laboratory experiment and 705.68 mV for HERMES III. These expected modulation voltages are well within the measurement range capability of a standard oscilloscope. In the device model, we considered the applied MITL voltage to be determined from the radially directed electric field lines that span a distance (s = 14 cm) and impinge perpendicularly on the surface of the crystal sensor. The length of the LiNO_3_ sensor (10 mm) is significantly less than the circumference of the cylindrical MITL, and therefore we do not incorporate curvature or field edge effects in the electric field calculation. The expected peak applied external electric field **E**_**a**_ (or V_a_/s) for the laboratory and HERMES III experiment are 6.32 kV/cm and 116 MV/m with corresponding applied voltages of 4.2 kV and 16.25 MV respectively.

### EOS laboratory and HERMES III experiment description

**Figure 1:**
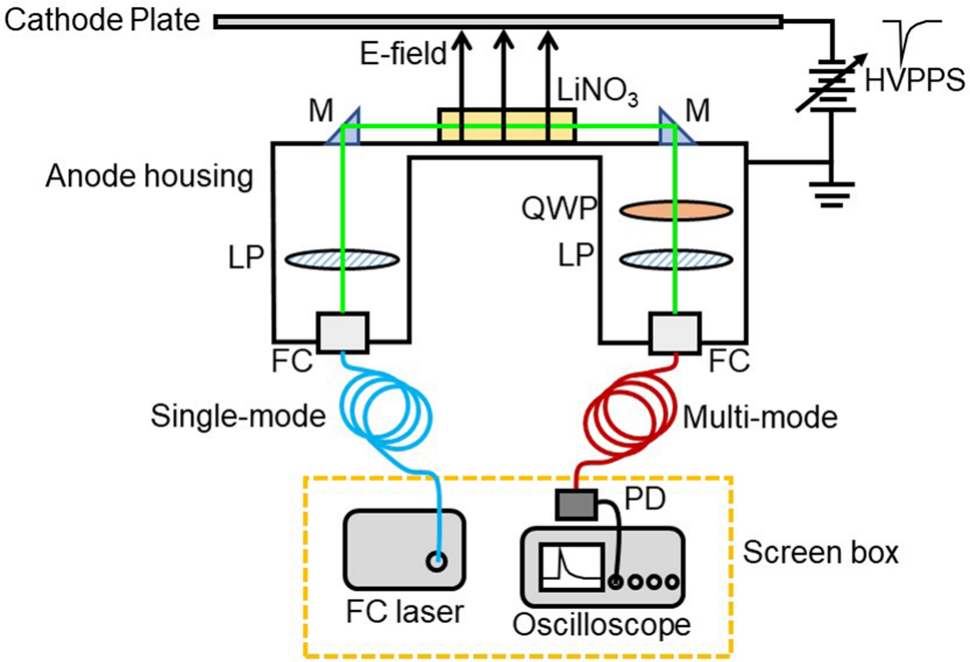
A schematic diagram and picture of the EOS benchtop laboratory setup including the 532 nm fiber coupled laser, high pulsed power supply (HVPPS) connected to the cathode plate and EOS anode housing, fiber coupling (FC) lenses, nano-particle coated linear polarizers (LP), lithium niobate crystal, right-angle mirrors (M), quarter waveplate (QWP), optical fibers, and fiber coupled photodetector (PD).

The main components of the EOS consisted of a 250-mW (reduced to 125 mW) continuous wave, single transverse mode, low-noise 532-nm fiber-coupled laser, a high-speed (1 GHz) silicon photodetector and a custom designed EOS housing to contain the 10 mm by 6 mm by 6 mm lithium niobate crystal and the high extinction ratio (10,000:1) nanoparticle coated linear polarizers and quarter waveplate. Lithium niobite is an ideal material for the EOS crystal as it has a relatively high electro-optical coefficient and durable physical properties. The EOS is designed to allow independent in situ adjustment of the optical beam alignment, light polarization and phase within the contained housing. The 532-nm laser light was coupled into a 25-meter long single-mode fiber where it is then directed through a set of right-angle mirrors, crossed polarizers, the EOS crystal and quarter waveplate and then coupled out of the EOS into a 25-meter long multi-mode fiber to the high-speed photodetector. The right-angle mirrors direct the light through the EOS and serve to protect the lithium niobate crystal from laterally directed energetic particle bombardment in the HERMES III experiment. The high polarization extinction ratio of the crossed polarizers enhances the signal-to-noise ratio and serves as a reference for the optical signal level. For the laboratory benchtop test, we placed the EOS next to a 38 mm diameter circular metal plate with a bias of 4.2 kV applied to the plate, and then used the metal body of the sensor as the ground plane as shown in Fig. 1.

**Figure 2:**
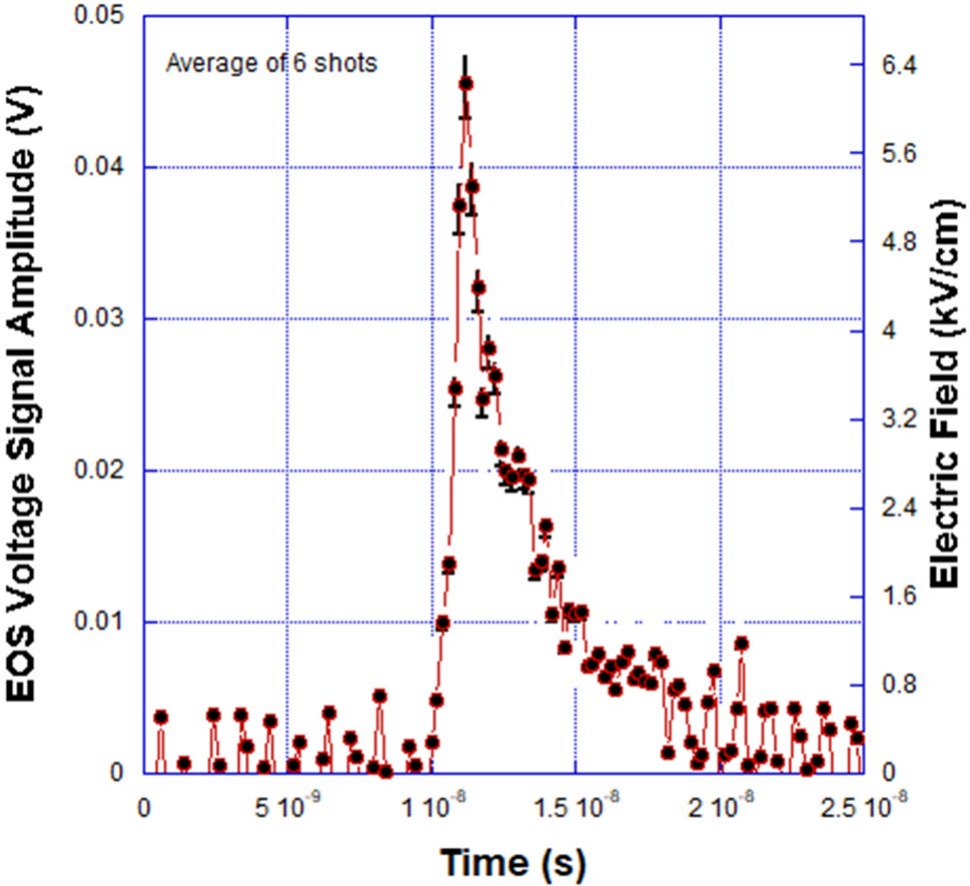
Benchtop laboratory EOS signal amplitude versus time for a series of six shots. The average peak pulse amplitude and electric field were 45.46 mV and 6.26 kV/cm compared to the expected values of 46.21 mV and 6.32 kV/cm from the calculation. The average pulse width for the EOS signals was 2.11 ns.

From the peak amplitude of the electro-optical signal generated directly from the electric field inside the EOS crystal, and the known operating parameters of the system, it is possible to determine the electric field and therefore the applied voltage bias in air or vacuum. A plot of the benchtop laboratory experiment modulation voltage generated from the electro-optical signal is shown in Fig. 2. A detailed description of the properties of the 4.2 kV high voltage supply driving pulse is provided in Owens et al.^7^. The average peak voltage and applied external electric field value for the laboratory experiment, which is shown to be 45.46 mV and 6.26 kV/cm in the graph, is in agreement with the device modeling prediction of 46.21 mV and 6.32 kV/cm described earlier. The benchtop experiment electric field signal-to-noise ratio (SNR) and resolution were approximately 15.5 dB and 140 V/cm respectively.

**Figure 3:**
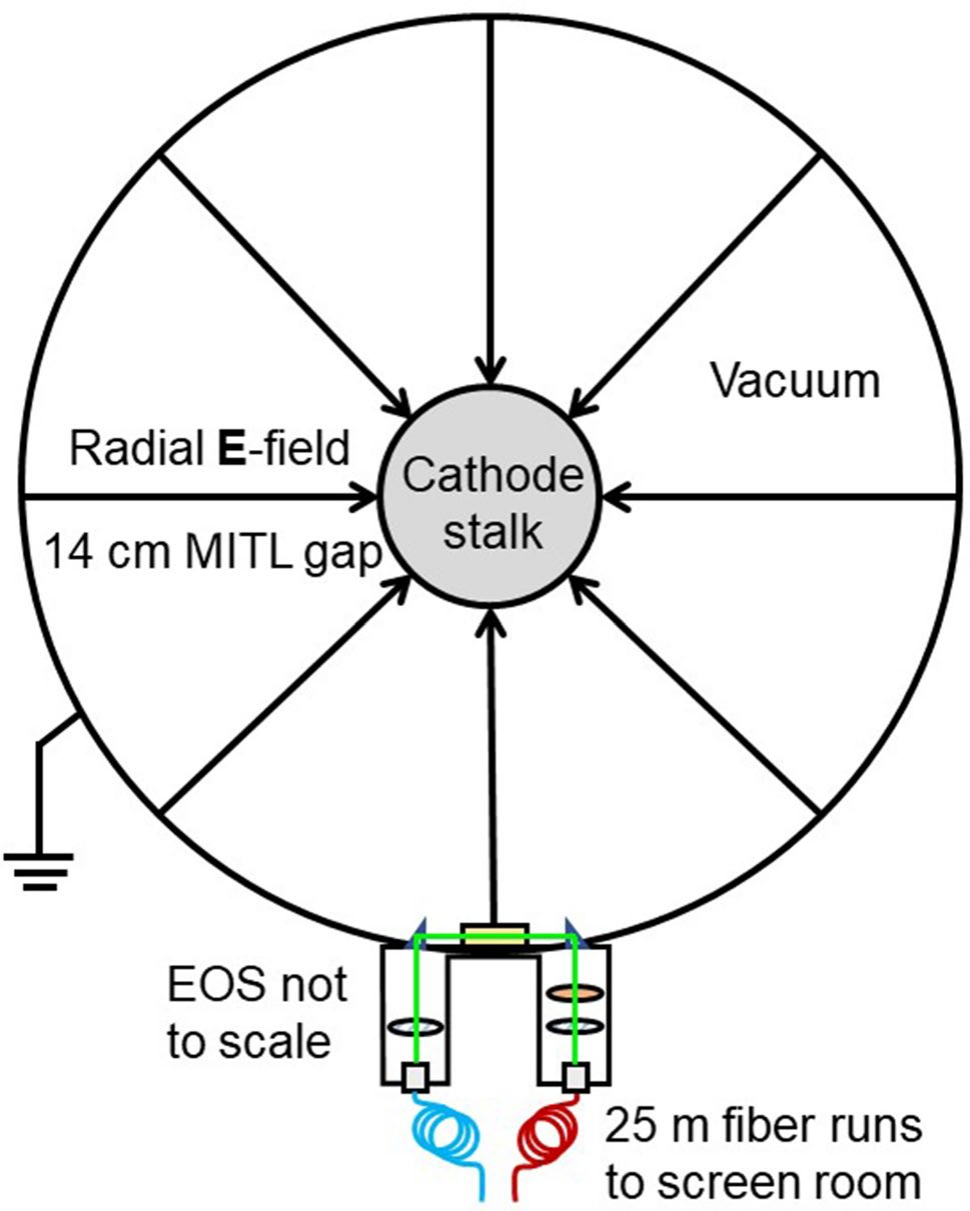
A schematic diagram of the EOS installed in the MITL region of the HERMES III pulsed power accelerator.

For the HERMES III experiment, the essential device experimental parameters were the same as the laboratory experiment, but the EOS was placed in the harsh environment of a high energy pulsed power accelerator MITL and exposed to substantially higher electric fields, electron bombardment and particle radiation. The high electric field is ultimately applied across a vacuum diode and used to accelerate electrons into a metal target, thereby producing Bremsstrahlung radiation (gamma rays) with energies up to 20 MeV. In considering the environment challenges, the EOS was very carefully designed with minimal sensing components and material protrusion into the radial gap between the MITL surface and ground as shown in Fig. 3.

The EOS sensing surface was aligned perpendicular with the axis of the MITL and positioned flush with the MITL anode’s inner surface, placing it approximately 14 cm from the cathode stalk. The side region of the MITL is ideal for EOS placement as it is out of the direct path of the electron beam and gamma radiation. In this region, however, the EOS experiences a higher electric field since the 14 cm radial gap is shorter than the nominal 58 cm anode to cathode (AK) gap in the vacuum diode at the end of the cylindrical MITL stalk. As with the benchtop laboratory experiment, 532 nm light was coupled and directed through the EOS via long optical fibers. The laser, photodetector and measurement instrumentation were housed inside of a remote Faraday cage screen room. When the HERMES III accelerator shots were fired, a very high electric field was generated between the MITL cathode stalk and anode which was measured by the EOS. Since the geometry of the MITL is fixed, it is therefore possible to determine both the electric field and applied voltage. The MITL is surrounded by trapped electron flow often referred to as an electron sheath^18^. With the electron sheath, the MITL operational impedance is slightly less than the local vacuum impedance^19^. However, its effect on the electric field measurement is negligible since the mm scale thickness of the sheath^18^ is short compared to the radial gap size.

## EOS results and discussion

**Figure 4:**
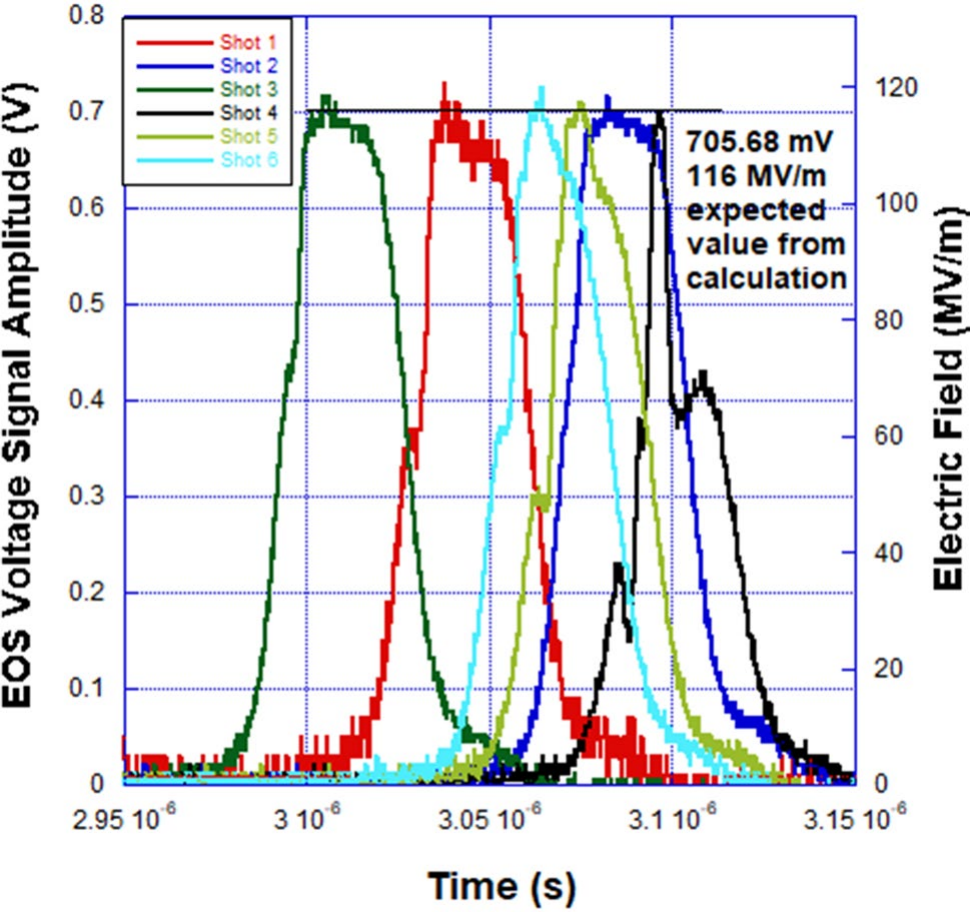
EOS signal amplitude versus time for a series of six shots on the HERMES III pulsed power accelerator. The average peak pulse amplitude and electric field were 715.66 mV and 117.86 MV/m compared to the expected values of 705.68 mV and 116 MV/m from the calculation. The average pulse width (excluding shot #4) for the EOS signals was 33.30 ns.

**Figure 5:**
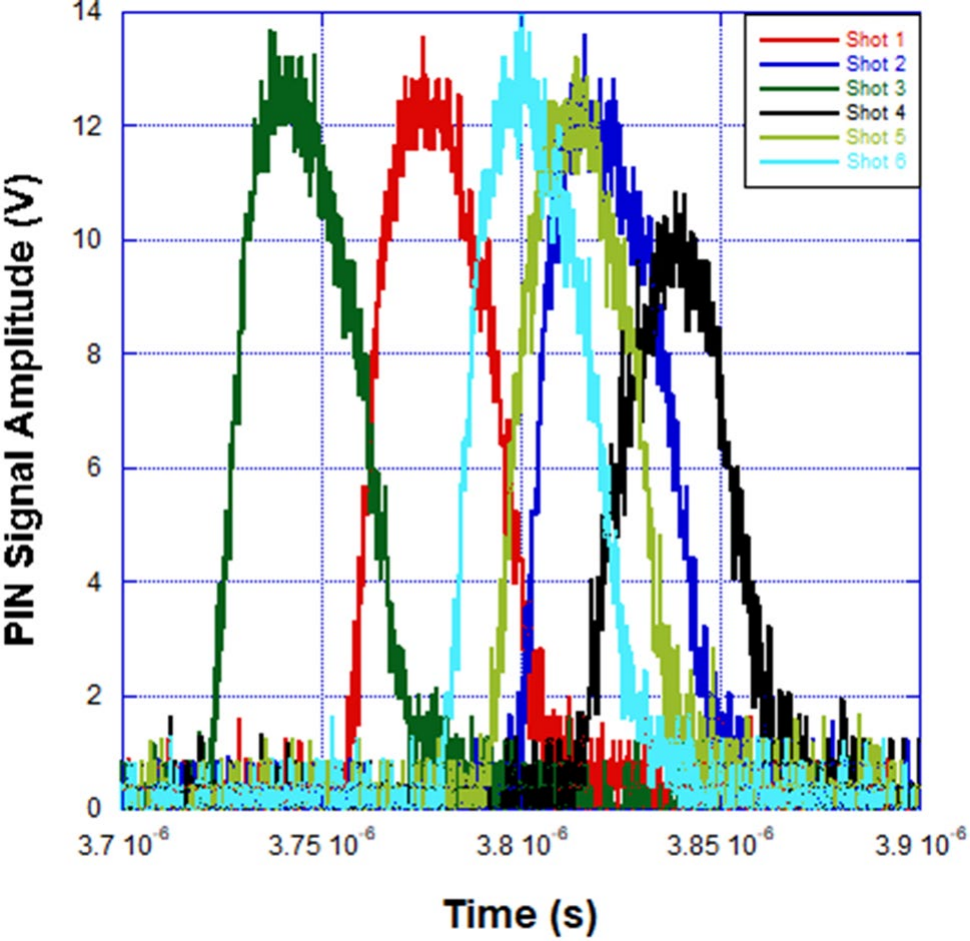
PIN diode signal amplitude versus time for a series of six shots on the HERMES III pulsed power accelerator. The average signal amplitude and pulse width (excluding shot #4) were 13.73 V and 32.24 ns respectively.

**Figure 6:**
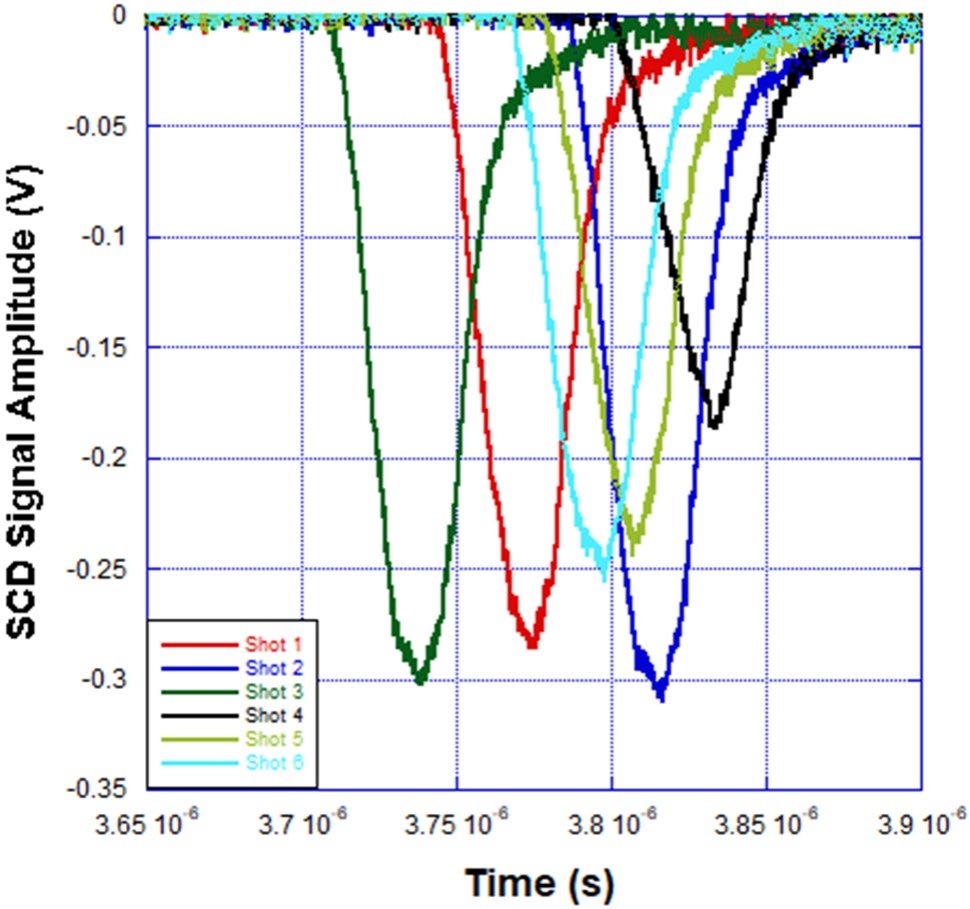
SCD signal amplitude versus time for a series of six shots on the HERMES III pulsed power accelerator. The average signal amplitude and pulse width (excluding shot #4) were -280.67 mV and 31.64 ns respectively.

In the discussion of the EOS waveforms that follow, we have included the model calculations, benchtop laboratory results and the responses generated by two radiation detectors in the HERMES III test cell—a PIN diode and spherical Compton diode (SCD)—that were monitored during the HERMES III experiment. During X-ray and gamma irradiation of a PIN diode detector, electron–hole pairs are generated within the silicon. This flow of carriers constitutes a photocurrent that can be measured in response to a radiation pulse. SCDs^20^ are energy resolving detectors and provide an electrical signal related to the production of secondary electrons (mostly Compton scattered electrons) following the interactions of the incident radiation with the diode. The PIN diode and SCD were positioned 10.5 m and 40 cm in front of the radiation converter, respectively. The PIN was biased to -210 V, while the SCD does not require an external bias and can withstand close placement relative to the converter and high irradiation levels without sustaining any physical damage. The voltage output of the PIN diode and SCD detectors show the general temporal behavior of the HERMES III radiation source, but neither detector has been calibrated to provide a quantifiable radiation dose. Plots of the modulation voltage for the EOS, and the voltage output from the PIN diode and the SCD are shown versus time in Figs. 4, 5 and 6.

From the plot of EOS signal amplitude versus time shown in Fig. 4, it is demonstrated that the average peak amplitude and applied external electric field of 715.66 mV and 117.86 MV/m again agree very well with the model calculation value of 705.68 mV and 116 MV/m. The HIII experiment electric field SNR and resolution were approximately 29.5 dB and 165 kV/m respectively. The peak amplitudes and external electric field values are uniform (except for “Shot 4”) over the six HERMES III shots in the series. The calculated and measured modulation voltages and electric fields correspond to radial MITL voltages of 16.25 MV to 16.5 MV. This span is within 1.75% difference and in agreement with previously estimated accelerator MITL voltages using data from conventional pulsed power diagnostics in the region where the EOS was installed^21^. In Figs. 5 and 6, it is shown that both the PIN and SCD produced pulses with uniform FWHM and peak amplitude values, again with the exception of “Shot 4”, for the shot series. Even though the PIN and SCD were external to the MITL electric field region and detected the radiation signal, the pulse width for both detectors were within 5.25% difference in value compared to the EOS. The electro-optical pulse produced by the EOS accurately reproduced the qualitative and quantitative features of the electric field waveform in the MITL of HERMES III. The EOS also reproduced higher bandwidth features of the MITL electric field waveform (e.g. “Shot 4”) that were more crudely detected and recorded as reduced amplitude radiation waveforms in the PIN and SCD detectors.

In addition to the results for the qualitative and quantitative aspects of the waveforms, the EOS showed robust performance in a challenging operating environment. During the experiment, there was no sign of outgassing from the sensor materials or any decrease in quality of the usual vacuum space. Furthermore, the side mirror protected the crystal from the possibility of direct lateral electron bombardment in the MITL during the rise and fall of the voltage pulse, the high internal field inside the crystal did not lead to dielectric breakdown, and there was no evidence of any electrical arcing on the surface. The optical fibers were outside of the vacuum chamber and positioned far away from the radiation source such that radiation darkening did not affect the light inside the optical fiber. Lithium niobate does have a notable acoustic response^22^. However, acoustical optical responses due to mechanical vibrations or similar effects caused by changes in temperature occur over a significantly larger time scale compared to the desired electro-optical signal, and had no interfering effect on the electric field measurement.

## Summary

In summary, an experiment to electro-optically measure an extremely high electric field with a relatively narrow pulse width in the MITL region of the HERMES III pulsed power accelerator was performed. We overcame numerous harsh environmental challenges in the region where the EOS was placed to perform the measurement, and the results agreed well with the theory, numerical calculations and an earlier benchtop laboratory experiment. To date, the results represent one of the highest overall external electric fields measured electro-optically in either a pulsed power accelerator or in related science experiments. Future interest includes exploring how various laser operating wavelengths, crystal materials and different pulsed power accelerator environments affect overall EOS operation and electric field measurement capability.

## Data Availability

Data is available by request from the authors.
